# Healthcare Costs and Workloss Burden of Patients with Chemotherapy-Associated Peripheral Neuropathy in Breast, Ovarian, Head and Neck, and Nonsmall Cell Lung Cancer

**DOI:** 10.1155/2012/913848

**Published:** 2012-03-14

**Authors:** Crystal T. Pike, Howard G. Birnbaum, Catherine E. Muehlenbein, Gerhardt M. Pohl, Ronald B. Natale

**Affiliations:** ^1^Analysis Group, Inc., Boston, MA 02199, USA; ^2^Eli Lilly and Company, Indianapolis, IN 46285, USA; ^3^Cedars-Sinai Medical Center, Los Angeles, CA 90048, USA

## Abstract

*Objective*. Chemotherapy-associated peripheral neuropathy (CAPN) is a painful side-effect of chemotherapy. This study assesses healthcare and workloss costs of CAPN patients with breast, ovarian, head/neck, or non-small cell lung cancer (NSCLC) from a third-party payor/employer perspective. *Research Design and Methods*. Patients with qualifying tumors, and claims for chemotherapy and services indicative of peripheral neuropathy (PN) within 9-months of chemotherapy (cases) were identified in a administrative claims database. Cases were matched 1 : 1 to controls with no PN-related claims based on demographics, diabetes history and propensity for having a diagnosis of PN during the study period (based on resource use and comorbidities in a 3-month baseline period). Average all-cause healthcare costs, resource use and workloss burden were determined. *Results*. Average healthcare costs were $17,344 higher for CAPN cases than their non-CAPN controls, with outpatient costs being the highest component (with cases having excess costs of $8,092). On average, each CAPN case had 12 more outpatient visits than controls, and spent more days in the hospital. Workloss burden was higher for cases but not statistically different from controls. *Conclusion*. This study establishes that breast, ovarian, head/neck, or NSCLC patients with CAPN have significant excess healthcare costs and resource use.

## 1. Introduction

Chemotherapy-associated peripheral neuropathy (CAPN) is a neurological side effect of chemotherapy characterized by loss of sensation in the hands and feet, burning or tingling in limbs, and, in some cases, loss of hearing and blurred vision. The neuropathic symptoms are progressive and tend to increase as chemotherapy treatment proceeds. In addition, comorbid conditions may exacerbate the severity of CAPN [1]. For example, diabetes can lead to peripheral neuropathy, and diabetic patients with pre-existing nerve damage may be predisposed to more severe forms of CAPN [2–4].

The chemotherapy drugs most commonly associated with CAPN are taxanes (paclitaxel and docetaxel), vinca alkaloids (vincristine and vinorelbine), and platinums (cisplatin, carboplatin, and oxaliplatin). The incidence of CAPN varies by drug and dose and can range across products from 4–92% [1, 2, 5, 6]. For example, clinical trials of paclitaxel in breast cancer list incidence rates for severe CAPN between 2–33%, with overall CAPN rates upwards of 60% [3, 7, 8]. Currently, there are no standard treatments to prevent or mitigate CAPN, although several drug classes (e.g., tricyclic antidepressants, antiepileptic drugs, and adjuvant analgesics) have shown some activity in reducing neuropathic pain [9, 10].

Few data exist regarding the health outcomes of CAPN patients, CAPN's effects on chemotherapy treatment, and associated costs. However, Berger suggests neuropathies in general can lead to adverse outcomes and higher costs [11]. In particular, Berger found that patients with neuropathies had healthcare costs triple those of controls; however, the study did not examine the costs associated with chemotherapy-related neuropathies specifically [11].

Calhoun conducted a pilot study on the medical and workloss costs associated with chemotherapy-induced toxicities in women with ovarian cancer [12]. Using survey data on 42 patients suffering from chemotherapy-induced neurotoxicities, the study found the medical costs directly attributable to CAPN were $688 per episode but that indirect costs (patient and caregiver workloss and paid caregiver costs) were over $4,200 per episode. This pilot study relied on patient recall of medical services used over 3-month intervals. In addition, the sample was limited to women with ovarian cancer and did not consider other cancer types.

To the authors' knowledge, no study has quantified the comprehensive health outcomes, medical costs, and workloss burden of CAPN patients with breast, ovarian, head/neck, or nonsmall cell lung cancer (NSCLC). The purpose of the current study is to assess health outcomes as well as the healthcare (i.e., medical and drug) and workloss cost burden of CAPN patients (cases) in these 4 tumor types from a third-party payor/employer perspective. The first objective is to compare the healthcare costs of CAPN cases with those of matched controls who have the same cancer but no CAPN. The second objective is to compare workloss costs in patients with and without CAPN. The third objective is to compare the healthcare costs of CAPN cases and non-CAPN controls who have comorbid diabetes. By examining these 4 tumor types, this study captures the use of the chemotherapeutic agents most commonly associated with CAPN.

## 2. Methods

### 2.1. Data

Data were obtained from a database of privately insured administrative claims records (Ingenix Employer Database) that included approximately 8 million beneficiaries from 40 large US-based companies (1999–2006). The companies have operations nationwide in a broad array of industries and job classifications. The database contains de-identified beneficiary information including demographics (e.g., age and gender), enrollment, and medical and pharmacy claims. Utilization measures include date of service, diagnoses, procedures, and actual payments to providers. Pharmaceutical drug claims include National Drug Code (NDC), fill date, days of supply, quantity, and actual payments. In addition, disability claims and employee wage information were available for employees in 23 companies.

### 2.2. Sample Selection

Three analytic samples consisting of CAPN cases and matched non-CAPN controls were used for this study. The main sample, consisting of cases and controls without diabetes, was used to evaluate the healthcare costs and resource use associated with CAPN patients. A sample of cases and controls with diabetes was used in the secondary analysis to evaluate the costs and resource use associated with diabetic CAPN patients. Finally, a subsample of employed cases and controls was used to assess the workloss burden associated with CAPN patients.  Table 1(a) presents the sample selection.

#### 2.2.1. Main CAPN Sample

Patients under age 65 were eligible for inclusion in the main sample if they had at least 1 claim with a diagnosis for 1 or more of the following cancers from 1999–2005: NSCLC, breast, ovarian, or head and neck. The cancer types were identified using ICD-9-CM codes (see Table 1(b)). To identify NSCLC from the overall lung cancer sample, patients receiving chemotherapy regimens characteristic of treatment for small cell lung cancer (SCLC) were excluded. SCLC treatment was defined in this study as doublet therapy with a platinum agent in combination with irinotecan, topotecan, or etoposide, or CCNU, melphalan, and VP-16 CAV regimen chemotherapy treatments. Of the patients with a claim for a qualifying tumor, only those with a procedure code indicating chemotherapy administration within 3 months of a claim for a qualifying tumor were selected. The date of first such chemotherapy administration was considered the index date. To ensure that the index event marked the start of a new line of chemotherapy for the tumor, patients were required to have at least 3 months of continuous eligibility prior to the index date with no claims for chemotherapy. Since no specific diagnosis code exists for CAPN, the authors developed an algorithm to define peripheral neuropathy (PN) using ICD-9-CM codes for related diagnoses and symptoms (see Table 1(b)). Any PN defined in the 9 months following the index date was assumed to be CAPN. Thus, patients were classified as CAPN if they had evidence of PN within 9 months of first chemotherapy treatment but had no evidence in the 3-month baseline period. Finally, for this sample, patients with evidence of diabetes (i.e., a diagnosis of 250.x) from at least 3 months up to 12 months prior to the index date or during the 12 months following the index date were excluded.

#### 2.2.2. Diabetic CAPN Sample

A sample of patients with 1 of the 4 tumor types, CAPN, and diabetes were selected for the secondary analysis. The patient selection criteria were the same as in the Main CAPN sample with the exception of the diabetes criteria. Specifically, diabetic CAPN patients were identified as those patients with a diagnosis of ICD-9-CM code 250.x anytime from at least 3 months up to 1 year prior to the index date or during the 12 months following the index date.

#### 2.2.3. CAPN Employee Sample

A subsample of patients from the Main CAPN and Diabetic CAPN samples who were employees with disability data were selected for the workloss cost analysis. Note that this sample will not contain all employed persons from the main and diabetic CAPN samples but rather will only include the employees of the subset of companies with disability data available.

#### 2.2.4. Study Period for All Samples

The study period encompassed the 12 months following the index date. Since the perspective adopted was that of the payor and the payor incurs no cost for patients who withdraw from coverage, no requirements were placed on the length of continuous eligibility following the index event. The baseline period for assessing patient history extended 3 months prior to the index date except for ascertaining the presence of diabetes. In all cases, the patients were required to have a minimum of 3 months eligibility prior to the index date to qualify for the study.

### 2.3. Matching

Cases in the main CAPN sample and the diabetes CAPN sample were separately matched to controls selected from among the set of NSCLC, breast, ovarian, and head/neck cancer patients receiving chemotherapy who did not have a diagnosis for CAPN-related symptoms at any time in the claims history and were under age 65. Controls were matched to cases based on age, gender, employment status (employee versus nonemployee), cancer type, index date of chemotherapy, length of followup (controls were required to have postindex eligibility of at least as long as their matched case), and the estimated likelihood of developing CAPN. Each case was matched 1 : 1 to a control using an optimal matching algorithm [13]. Likelihood of developing CAPN was derived as a propensity score from a logistic regression model based on resource use and comorbidities during the baseline period. Specifically, the model included age, sex, Charlson Comorbidity Index (CCI) [14], number of inpatient, primary care, oncology, neurology, and other physician visits, and binary variables indicating whether patient went to the emergency room or had depression or uncomplicated hypertension.

The time period over which controls contributed to the outcomes measures was truncated at the end of the observation period for their matching case (i.e., the earlier of either 12 months or when cases dropped from the database).

### 2.4. Measures

Healthcare costs were stratified into 3 mutually exclusive groups: chemotherapy costs, drug costs, and medical costs. Costs were computed as the paid (reimbursed) amount by the insurer to the health-care provider and were annualized to 2006 U.S. Dollars using the Consumer Price Index for medical care. Chemotherapy costs included medical claims with a chemotherapy procedure code and pharmacy claims for oral chemotherapy agents, identified by NDCs. Drug costs included all pharmaceutical claims other than chemotherapy claims. Medical costs included claims for inpatient, emergency department (ED), and outpatient/other care. Costs included all claims associated with any service provided to the case/control during the study period regardless of diagnosis, procedure, or drug. The subset of pharmaceutical and medical costs that could be directly attributed to CAPN was estimated as follows: CAPN-related drug costs included those for drugs potentially used for CAPN (i.e., amitriptyline, gabapentin, amifostine, glutamine, tricyclic antidepressants, anti-epileptics, NSAIDs, and opioids) and CAPN-related medical costs included those from claims with a primary or secondary diagnosis of a CAPN-related symptom (see Table 1(b)).

Healthcare resource use consisted of hospitalizations, emergency department (ED), and outpatient/other services (reported by type of visit). The resource use components were defined using provider specialty codes and/or place of service codes on the claims. All claims during the study period were included in assessing resource use, regardless of the underlying reason for a visit. In addition, the proportion of patients using CAPN-related drugs, and specific chemotherapy agents were measured.

Workloss days and costs consisted of disability and medically related absenteeism. Workloss costs during the 12-month study period included actual employer payments for disability days plus imputed costs for medically related absenteeism. Medically related absenteeism costs were imputed by multiplying the number of days with medical services resource use by the employee's wage: each hospitalization day accounted for a full day of workloss, and outpatient visits accounted for half a day of workloss. As with healthcare costs, total workloss costs were not limited to only those related to a particular condition.

Patient characteristics included demographics, employment status (employee versus nonemployee), cancer type, cancer stage (metastatic versus not metastatic), and comorbidities identified using claims during baseline period and the 12-month study period. Metastatic cancer was defined using claims with diagnoses for metastases (ICD-9-CM codes 196.0-199.1). The CCI was calculated from the claims data and individual physical comorbidities included in the index which were also identified [14]. 

### 2.5. Statistical Analyses

Baseline characteristics (demographics, comorbidities, resource use rates) were summarized as proportions of the sample with the characteristic. Continuous measures (e.g., healthcare costs, workloss costs, resource use amount) were summarized by mean and standard deviation. Comparisons of matched pairs of categorical variables used McNemar tests. Comparisons of the differences in continuous measures between cases and controls used paired *t*-tests. Excess costs of CAPN cases compared with controls (i.e., costs of CAPN cases minus those of controls) were compared between diabetic and nondiabetic patients using a 2-sample *t*-test. All analyses were conducted using SAS version 9.1 (SAS Institute Inc., Cary, NC). *P*-values less than or equal to 0.05 were considered statistically significant.

## 3. Results

The main study sample for the healthcare cost and resource use analysis contained 454 cases and controls without diabetes (see Table 1(a)). The diabetes sample for the secondary analysis contained 71 diabetic cases and controls. The employee subsample for the workloss cost and resource use analysis contained 78 cases and controls.

### 3.1. Baseline Characteristics

Tables 2(A) and 3(A) show the baseline comparison of CAPN cases and their matched non-CAPN controls within the main sample. Cases and controls were balanced in terms of types of cancer and CCI. However, CAPN cases had more congestive heart failure (4% versus 2%, *P* = 0.0412) and uncomplicated hypertension (17% versus 11%, *P* = 0.0164), whereas the control group had a higher rate of complicated hypertension (3% versus 1%, *P* = 0.0290). Resource use was generally the same between cases and controls; however, there were more cases with neurology specialist visits than controls (5% versus 2%, *P* = 0.0482). The number of neurology visits and neurology costs were not significantly different.

### 3.2. Study Period Descriptive Characteristics, Healthcare Resource Use, and Costs

CAPN cases had significantly higher rates and counts of comorbidities and resource use during the study period compared with matched non-CAPN controls (Tables 4(A) and 5(A)). Significantly more cases had fibromyalgia, obesity, and uncomplicated hypertension during the study period than their matched controls. Cases also had a higher CCI during the study period (4.7 versus 4.1, *P* < 0.0001). There were no differences in the classes of chemotherapy agents used by cases and controls. The most common chemotherapeutic agents used by cases and controls were taxanes (33%, 28%) and platinums (20%, 18%). Significantly more cases used a CAPN-related drug than controls (72% versus 56%, *P* < 0.0001). Cases had substantially higher rates and amounts of use of both inpatient and outpatient visits during the study period. More cases were hospitalized at least once compared with controls (51% versus 37%, *P* < 0.0001). CAPN cases had significantly higher rates and amounts of use for all outpatient components except for primary care visits and lab/pathology. More cases saw a neurologist than did controls (29% versus 6%, *P* < 0.0001).

Table 6(A) shows the healthcare cost comparison for cases and controls. For cases, mean annual per capita healthcare costs were $69,950 versus $52,606 per control, with an excess annual per patient cost of $17,344 (*P* < 0.0001). Mean excess annual per patient healthcare costs for cases versus controls were $36,660 for head and neck cancer, $18,790 for nonsmall cell lung cancer, $16,940 for breast cancer, and $5,140 for ovarian and all were statistically significant (data not shown). Cases had significantly higher component costs compared with controls. Outpatient costs were the highest component for both cases and controls. However, the excess costs of outpatient and inpatient components were similar with cases having excess annual outpatient costs of $8,092 per patient (*P* < 0.0001) and excess annual inpatient costs of $7,552 per patient (*P* < 0.0001). Annual oncology-related costs (i.e., chemotherapy and oncologist specialist costs) were $22,453 for cases compared with $19,362 for controls, with the majority of costs being chemotherapy costs ($16,984 and $16,169 for cases and controls, resp., *P* = 0.5744). CAPN-related drug and medical costs accounted for approximately 2% of total healthcare costs.

### 3.3. Workloss and Costs

There were no statistically significant differences in workloss measures between the subset of 78 employees with CAPN and their matched non-CAPN controls (Table 7). More cases had disability claims than controls (35% versus 26%, resp.). Cases also had almost twice as many disability days as their matched controls (37.4 versus 20.4 days, resp.). While almost all cases and controls had at least 1 medically related absenteeism day (95% and 97%, resp.), cases missed 6 additional days over the 12-month study period compared with controls.

Average annual workloss costs were approximately 25% higher for cases than controls ($11,298 versus $9,043, resp.) with a $2,255 annual per patient difference (Table 7). Cases had both higher disability and higher medically-related absenteeism costs than controls ($4,970 versus $3,356 for disability and $6,329 versus $5,687 for medically-related absenteeism). However, workloss cost differences were not statistically significant.

### 3.4. Secondary Analysis of CAPN and Diabetes

Tables 2(B) and 3(B) show the baseline comparison of diabetic cases and their matched diabetic controls. Cases had more metastatic disease (46% versus 28%, *P* = 0.0374), complicated hypertension (6% versus 0%, *P* = 0.0290), and a higher CCI than controls (6.0 versus 4.5, *P* = 0.0043). Resource use was generally well balanced; however, cases did have more primary care visits than controls (2.75 versus 1.75, *P* = 0.0136). There were no statistically significant differences in baseline costs.

Cases had significantly higher rates and counts of resource use during the study period compared with controls (see Tables 4(B) and 5(B)). More cases than controls used taxanes (42% versus 24%, *P* = 0.0236). More cases were hospitalized compared with controls (68% versus 45%, *P* = 0.0136) though the days per capita were not statistically different (9.1 versus 6.2 for cases and controls, resp., *P* = 0.2195). Six times as many cases had a neurology specialist visit during the study period compared with controls (42% versus 7%, *P* < 0.0001). Cases also had 18.4 more outpatient visits during the study period on average than the controls (*P* < 0.0001). CAPN-related drugs were used by 93% of cases compared with 65% of controls (*P* = 0.0003).

For cases, annual per capita healthcare costs were $76,555 versus $54,816 per control, with an excess annual per patient cost of $21,739 (*P* = 0.0273, Table 6(B)). Annual oncology-related costs were $25,181 for cases compared with $15,377 for controls, with the majority of these costs being chemotherapy costs ($20,990 and $13,033 for cases and controls, resp., *P* = 0.0670). The diabetic case's annual per capita excess costs were higher than the control's costs ($21,739 versus $17,344), however, this difference was not statistically significant.

## 4. Discussion

This study is the first to use claims data to estimate the excess costs of CAPN patients in breast, nonsmall cell lung, ovarian, and head and neck cancer over a matched sample of cancer patients without CAPN. This study also included a secondary analysis to examine the excess costs of CAPN patients among patients with comorbid diabetes.

The results suggest that CAPN patients are associated with a significant and substantial economic burden among the privately insured U.S. population. Cases with CAPN had on average $17,344 higher healthcare costs during the 12-month study period compared with controls without CAPN. Compared with controls, more cases were hospitalized, had an emergency department visit, saw an oncologist or neurologist, and had other outpatient visits. In addition, each case with CAPN averaged 12 more outpatient visits and spent more days in the hospital. This suggests that in addition to the excess cost burden to third-party payors, the patients themselves may (depending on their insurance benefits) experience a large burden in terms of out-of-pocket costs (e.g., copayments, coinsurance) and time spent on medical care.

CAPN can lead to increased costs as a result of services specifically aimed at mitigating the PN (e.g., increased physician visits to monitor the PN, costs of PN treatment) and secondary effects, such as switches in chemotherapy regimens or exacerbated cancer. This study separately estimated the costs for CAPN-related services and found they accounted for less than 2% of healthcare costs during the 12-month study period. While a significantly higher proportion of CAPN patients did use the pharmacologic treatments used to manage neuropathic symptoms, these drugs are often generic or lower cost relative to cancer treatments and overall healthcare costs. CAPN-related medical costs were low suggesting that the excess utilization demonstrated by cases was not directly attributed to CAPN. However, the CAPN-related costs as measured here may understate the true burden related specifically to CAPN. First, because there are no specific diagnoses for CAPN, PN-related diagnoses may be underreported on claims. In addition, the secondary effects of CAPN mentioned above are not included in the CAPN-related subset of costs presented here but rather are included in the chemotherapy, drug, and medical costs.

Overall, this study finds that patients with CAPN experience significantly increased costs and resource use. It is important not to conclude that all of the excess costs are caused by CAPN. Since the cases and controls were matched during the baseline period, the results suggest that CAPN is a serious condition that should be carefully monitored in clinical practice. Improvements to the diagnostic tools for CAPN severity and investigation of therapies that treat CAPN without negatively impacting the cancer treatment could benefit both patients and payors.

Calhoun's pilot study of chemotherapy-induced toxicity found that the direct CAPN costs for women with ovarian cancer are $688 for episodes up to 9 months, compared with our findings that CAPN-related costs are on average $1,320 over a period up to 12 months [12]. Calhoun's study relied on patient reported utilization and standard fee data whereas data here are actual reimbursements, which may, in addition to the difference in length of followup, explain the differences in estimates. Calhoun also analyzed indirect costs based on national labor force, employment, and earnings data, including patient workloss, caregiver workloss, and paid caregiver costs. Calhoun found indirect costs were $4,220, with 67% of that due to caregiver workloss. Patient workloss costs were $620 per patient per episode compared with our findings of $642 per patient per year in excess medically related absenteeism costs. Though Calhoun did not include disability costs, this study found CAPN patients had mean excess per patient per year disability costs of $1,614. While the workloss findings were not statistically significant, this may be due to the small sample size. It is important to note that the workloss cost estimates reported in this study may differ from actual employer costs depending on the employer's paid time off policies.

Healthcare costs and resource use were calculated for diabetic CAPN patients as a secondary analysis as literature suggests that diabetes may exacerbate the risk and severity of CAPN. With the prevalence of diabetes increasing, it is important to assess how diabetic CAPN patients may differ from controls and nondiabetic CAPN patients. The results demonstrated that diabetes may, indeed, be associated with increased cost in patients with CAPN, as excess costs of diabetic CAPN patients were 25% higher than for nondiabetic CAPN patients. However, the comparison between diabetic and nondiabetic CAPN patients was not statistically significant, perhaps due to the small sample of diabetic CAPN patients. In addition, although diabetic cases were balanced with their controls in terms of costs and resource use, a higher proportion of cases had metastatic cancer and the CCI of cases was significantly higher than controls. Both these factors could impact the excess cost findings.

This study is limited by the lack of clinical measures, which is common to research that uses claims data. While propensity score matching was used to adjust for many baseline group differences, the possibility exists of other confounding factors not available in this database. Moreover, because CAPN does not have a specific diagnosis code and is not consistently recorded in claims data, there are challenges in identifying the condition. Physicians may not report CAPN unless the impairment is severe enough to affect a patient's activities of daily living or warrant alterations to cancer treatment [1]. Thus, this study may include more severe cases of CAPN and may have under identified CAPN patients. The algorithm used for identifying CAPN in this study has not been validated. It is possible that the PN identified was due to other causes. Another study limitation is that due to data availability, cases and controls are matched using only 3 months of baseline data and information on cancer stage beyond metastatic/nonmetastatic is not available. A longer baseline period using a larger database with clinical information on cancer stage may allow for additional controls. The lack of restrictions on the length of continuous eligibility following index date is another possible limitation. This limitation could potentially lead to censoring of costs if a patient's plan were to have withdrawn from the database while the patient was continuing to incur costs; however, no such instances occurred in the study and the average length of followup for patients in the study is 11.1 months. Finally, small sample sizes, particularly in the head and neck, ovarian, workloss cost, and diabetic analyses, limited both the types of comparisons as well as the robustness of the statistical inferences. Though this study reports directional differences in workloss outcome measures, no significant differences were found, potentially due to the small sample size.

## 5. Conclusion

CAPN patients are associated with significantly higher healthcare costs and resource use in patients with breast, ovarian, head/neck, or NSCLC. The excess healthcare cost of CAPN is underestimated when only the cost for medical or pharmacy claims directly for CAPN is considered. Improvements in clinical assessments and treatments for CAPN would be useful for patients and payors.

## Figures and Tables

**Table:** (a) Inclusion criteria

	Criteria	Number of patients
1	Number of beneficiaries under 65 at eligibility start	4,729,443
2	>1 breast, ovarian, head and neck, or nonsmall cell lung cancer claim from 1999–2005	56,261
3	>1 chemotherapy treatment within 3 months following a breast, ovarian, head and neck, or nonsmall cell lung cancer diagnosis	14,142
4	>3 months of continuous eligibility prior to the first chemotherapy treatment for the qualifying tumor during which no other chemotherapy treatment was received	11,009
5	>1 diagnosis for peripheral neuropathy following first chemotherapy treatment	1,245
6	Peripheral neuropathy within 9 months of first chemotherapy treatment	525
	Main CAPN sample: no diabetes history	454
	Diabetic CAPN sample	71
	Employee CAPN subsample	78

**Table:** (b) ICD-9-CM Diagnosis Codes Used for Identification of CAPN and Cancer

CAPN	ICD-9-CM code
Polyneuropathy due to drugs	357.6
Disturbance of skin sensation	782.0
Inflammatory and toxic neuropathy	357.x
Toxic optic neuropathy	377.34
Reflex sympathetic dystrophy	337.2
Cervical root lesions	353.2
Lumbosacral root lesions	353.4
Other mononeuritis of unspecified site	355.7
Mononeuritis of unspecified site	355.9
Neuralgia, neuritis, or radiculitis	729.2
Brachial plexus lesions	353.0

Cancer	ICD-9-CM code

Nonsmall cell lung cancer	162.x
Breast cancer	174.x, 175.x
Ovarian	183.x
Head and neck	195.0, 140.x, 141.x, 142.x, 143.x, 144.x, 145.x, 146.x, 147.x, 148.x,149.x

**Table 2:** Three-month baseline demographics and comorbidities of CAPN cases and non-CAPN controls.

	(A) Main Sample: no diabetes					(B) Diabetes Sample				
	Cases		Controls		*P*-value	Cases		Controls		*P*-value^a^

	No.	%	No.	%		No.	%	No.	%	
*N*	454		454			71		71		
Demographics										
Age (mean, SD)	53.9	7.5	53.7	9.3	0.0006	58.5	5.0	57.9	8.3	0.5044
Gender (*n*, % male)	69	15%	69	15%	1.0000	13	18%	13	18%	1.0000
Employment Status (*n*, % employed)	75	17%	75	17%	1.0000	3	4%	3	4%	1.0000
Months followup (mean)	11.1		2.2			11.3		1.7		
12-month followup	366	81%				56	79%			
Time to CAPN (mean days, SD)	147.5	82.9				137.2	85.7			
1–3 months	125	27%				22	31%			
3–6 months	149	33%				26	37%			
6–9 months	179	39%				23	33%			

Cancer type										
Nonsmall cell lung	82	18%	82	18%	1.000	18	25%	18	25%	1.000
Breast	316	70%	316	70%	1.000	42	59%	42	59%	1.000
Ovarian	28	6%	28	6%	1.000	7	10%	7	10%	1.000
Head and neck	27	6%	27	6%	1.000	4	6%	4	6%	1.000

Metastatic cancer	168	37%	164	36%	0.7883	33	46%	20	28%	0.0374

Comorbidities										
Depressive disorders	23	5%	31	7%	0.2673	2	3%	3	4%	0.6547
Congestive heart failure	20	4%	10	2%	0.0412	7	10%	8	11%	0.7963
Fibromyalgia	7	2%	7	2%	1.0000	4	6%	1	1%	0.1797
Obesity	8	2%	2	0%	0.0578	2	3%	0	0%	0.0578
Hypertension—uncomplicated	76	17%	52	11%	0.0164	22	31%	24	34%	0.7316
Hypertension—complicated	4	1%	13	3%	0.0290	4	6%	0	0%	0.0290
Other cancers	83	18%	74	16%	0.4352	17	24%	21	30%	0.4497
Charlson Comorbidity Index	4.4		4.3		0.4913	6.0		4.5		0.0043

CAPN indicates chemotherapy-associated peripheral neuropathy; SD, standard deviation.

^a^ *P*-values are determined using McNemar tests for proportions and paired t-tests for continuous measures.

**Table 3:** Three-month per-capita baseline resource use and healthcare costs of CAPN cases and non-CAPN controls.

	(A) Main Sample: no diabetes					(B) Diabetes Sample				
	Cases		Controls		*P*-value^a^	Cases		Controls		*P*-value^a^

	No.	%	No.	%		No.	%	No.	%	
*N*	454		454			71		71		
Resource use rate										
Hospitalizations	204	45%	193	43%	0.4692	43	61%	33	46%	0.1138
ED visits	75	17%	68	15%	0.5139	21	30%	16	23%	0.3980
Outpatient visits	444	98%	445	98%	0.8185	71	100%	70	99%	0.8185
Oncology	188	41%	174	38%	0.3408	26	37%	30	42%	0.4795
Neurology	22	5%	11	2%	0.0482	5	7%	1	1%	0.1025

Resource use amount	Mean	(SD)	Mean	(SD)	*P*-value^a^	Mean	(SD)	Mean	(SD)	*P*-value^a^

Hospitalizations	1.96	(5.53)	1.48	(3.21)	0.0956	3.48	(5.02)	4.10	(9.75)	0.5702
ED visits	0.24	(0.70)	0.21	(0.58)	0.3550	0.41	(0.73)	0.35	(0.76)	0.6310
Outpatient visits	11.37	(6.80)	11.25	(7.40)	0.7449	12.80	(6.08)	11.28	(6.76)	0.1420
Oncology	1.40	(3.06)	1.33	(3.47)	0.6673	1.38	(3.10)	1.44	(2.97)	0.9040
Neurology	0.06	(0.31)	0.03	(0.22)	0.0848	0.07	(0.26)	0.01	(0.12)	0.1029

Total costs	$17,797	($22,255)	$17,180	($25,240)	0.6698	$22,308	($24,970)	$21,061	($22,748)	0.6898
Drug costs	$433	($857)	$467	($1,148)	0.6064	$800	($868)	$646	($824)	0.2761
Medical costs	$17,363	($22,185)	$16,712	($25,140)	0.6527	$21,508	($24,871)	$20,415	($22,636)	0.7268
Inpatient	$6,496	($19,301)	$5,928	($21,811)	0.6632	$11,220	($22,256)	$9,382	($21,412)	0.4845
ED	$127	($826)	$102	($576)	0.6043	$125	($290)	$122	($340)	0.9490
Outpatient	$10,336	($10,186)	$10,328	($10,282)	0.9900	$9,725	($8,672)	$10,358	($9,840)	0.6935
Oncology	$438	($1,329)	$439	($1,796)	0.9841	$379	($996)	$431	($1,452)	0.7989
Neurology	$10	($61)	$9	($108)	0.8812	$24	($163)	$1	($7)	0.2435

CAPN indicates chemotherapy-associated peripheral neuropathy; SD, standard deviation; ED, emergency department.

^a^ *P*-values are determined using McNemar tests for proportions and paired *t*-tests for continuous measures.

**Table 4:** Chemotherapy agents and comorbidities of CAPN cases and non-CAPN controls during the 12-month study period.

	(A) Main Sample: no diabetes					(B) Diabetes Sample				
	Cases		Controls		*P*-value^a^	Cases		Controls		*P*-value^a^

	No.	%	No.	%		No.	%	No.	%	
*N*	454		454			71		71		
Metastatic cancer	171	38%	136	30%	0.0097	30	42%	22	31%	0.1306

Classes of select chemotherapy agents used										
Taxanes	148	33%	125	28%	0.0978	30	42%	17	24%	0.0236
Vinca Alkaloids	18	4%	14	3%	0.4497	3	4%	2	3%	0.6547
Platinums	90	20%	80	18%	0.4014	17	24%	20	28%	0.5775

Comorbidities										
Depressive disorders	68	15%	50	11%	0.0804	8	11%	3	4%	0.1317
Congestive heart failure	23	5%	20	4%	0.6219	12	17%	12	17%	1.0000
Fibromyalgia	37	8%	8	2%	<.0001	8	11%	1	1%	0.0196
Obesity	9	2%	2	0%	<.0001	1	1%	0	0%	0.2568
Hypertension—uncomplicated	114	25%	83	18%	0.0134	33	46%	35	49%	0.7576
Hypertension—complicated	13	3%	13	3%	1.0000	5	7%	1	1%	0.1025
Other cancers^b^	124	27%	101	22%	0.0838	20	28%	19	27%	0.8415
Charlson Comorbidity Index	4.7		4.1		<.0001	7.0		5.6		0.0052

CAPN indicates chemotherapy-associated peripheral neuropathy.

^a^ *P*-values are determined using McNemar tests for proportions and paired *t*-tests for continuous measures.

^b^Other cancers include all cancers other than head and neck, breast, non-small cell lung, and ovarian.

**Table 5:** Resource use of CAPN cases and non-CAPN controls during the 12-month study period.

	(A) Main Sample: no diabetes					(B) Diabetes Sample				
	Cases		Controls		*P*-value^a^	Cases		Controls		*P*-value^a^

	No.	%	No.	%		No.	%	No.	%	
*N*	454		454			71		71		
Resource use rate										
Medical										
Hospitalizations	231	51%	166	37%	<.0001	48	68%	32	45%	0.0136
ED visits	213	47%	170	37%	0.0037	42	59%	27	38%	0.0222
Outpatient visits	453	100%	447	98%	0.0339	71	100%	70	99%	0.0339
Oncology	288	63%	250	55%	0.0075	43	61%	39	55%	0.5050
Neurology	133	29%	29	6%	<.0001	30	42%	5	7%	<.0001
Primary care	354	78%	345	76%	0.4726	59	83%	60	85%	0.8185
Other physician	444	98%	428	94%	0.0035	71	100%	66	93%	0.0035
Lab/pathology	181	40%	160	35%	0.1540	21	30%	22	31%	0.8694
Other outpatient	400	88%	369	81%	0.0030	68	96%	61	86%	0.0522

Prescription drug use										
At least 1 CAPN-related drug	329	72%	256	56%	<.0001	66	93%	46	65%	0.0003

Resource use amount	Mean	(SD)	Mean	(SD)	*P*-value^a^	Mean	(SD)	Mean	(SD)	*P*-value^a^

Medical										
Hospitalizations	5.6	(11.69)	3.2	(7.77)	0.0001	9.1	(14.38)	6.2	(13.44)	0.2195
ED visits	1.1	(2.80)	0.6	(1.28)	0.0022	1.6	(1.98)	0.8	(1.35)	0.0064
Outpatient visits	51.3	(29.57)	39.8	(26.81)	<.0001	56.9	(27.02)	38.5	(25.10)	<.0001
Oncology	12.7	(18.92)	9.2	(15.55)	0.0021	12.6	(16.97)	7.5	(10.62)	0.0322
Neurology	0.6	(1.29)	0.1	(0.51)	<.0001	1.1	(1.87)	0.1	(0.56)	0.0002
Primary care	6.9	(12.65)	5.0	(8.74)	0.0085	7.4	(10.25)	5.7	(5.64)	0.2454
Other physician	22.1	(21.22)	17.2	(18.82)	0.0001	25.9	(20.63)	18.6	(18.82)	0.0217
Lab/pathology	1.1	(2.62)	0.9	(2.49)	0.2127	0.8	(3.59)	1.1	(3.93)	0.6594
Other outpatient	13.6	(16.47)	11.4	(15.97)	0.0349	15.5	(15.37)	11.0	(16.56)	0.0905

CAPN indicates chemotherapy-associated peripheral neuropathy; SD, standard deviation; ED, emergency department.

^a^ *P*-values are determined using McNemar tests for proportions and paired *t*-tests for continuous measures.

**Table 6:** Per capita healthcare costs for CAPN cases and non-CAPN controls during the 12-month study period.

	(A) Main Sample: no diabetes					(B) Diabetes Sample				
	Cases		Controls		*P*-value^a^	Cases		Controls		*P*-value^a^

	mean	(SD)	mean	(SD)		mean	(SD)	mean	(SD)	
*N*	454		454			71		71		
Total healthcare costs	$69,950	($66,913)	$52,606	($55,554)	<.0001	$76,555	($63,379)	$54,816	($68,115)	0.0273

Chemotherapy costs	$16,984	($21,248)	$16,169	($27,055)	0.5744	$20,990	($31,501)	$13,033	($19,725)	0.0670

Drug costs	$3,744	($5,333)	$3,071	($4,927)	0.0419	$6,017	($7,461)	$4,223	($6,100)	0.1163
CAPN-related drugs	$595	($1,590)	$328	($1,041)	0.0016	$718	($1,401)	$371	($782)	0.0832

Medical costs	$49,223	($56,500)	$33,366	($36,931)	<.0001	$49,548	($46,428)	$37,561	($57,723)	0.1614
Inpatient	$14,050	($35,793)	$6,498	($15,558)	<.0001	$19,181	($38,190)	$15,148	($44,098)	0.5637
ED	$474	($1,093)	$263	($730)	0.0005	$798	($1,817)	$243	($535)	0.0172
Outpatient	$34,698	($36,712)	$26,606	($29,913)	<.0001	$29,569	($21,339)	$22,170	($29,244)	0.0921
Oncology	$5,469	($15,923)	$3,193	($8,478)	0.0072	$4,191	($6,950)	$2,344	($4,729)	0.0554
Neurology	$129	($319)	$46	($433)	0.0011	$326	($843)	$44	($278)	0.0103
Primary care	$7,772	($12,454)	$6,543	($9,920)	0.0979	$6,024	($7,341)	$4,472	($5,965)	0.1252
Other physician	$7,437	($13,782)	$6,804	($17,452)	0.5247	$6,235	($11,447)	$5,150	($16,025)	0.6502
Lab/pathology	$414	($1,825)	$289	($1,490)	0.2632	$485	($2,027)	$110	($193)	0.1237
Other outpatient	$13,476	($24,509)	$9,731	($18,345)	0.0064	$12,307	($16,297)	$10,049	($20,812)	0.4798
CAPN-related medical costs	$725	($2,005)				$491	($762)			

CAPN indicates chemotherapy-associated peripheral neuropathy; SD, standard deviation; ED, emergency department.

^a^ *P*-values are determined using McNemar tests for proportions and paired *t*-tests for continuous measures.

**Table 7:** Per capita workloss days and costs during the 12-month study period.

	Employee subsample				
	Cases		Controls		*P*-value^a^

	No.	%	No.	%	
*N*	78		78		

Workloss rate^b^					
Medically related absenteeism	74	95%	76	97%	0.4142
Disability	27	35%	20	26%	0.2367

Workloss days	mean	(SD)	Mean	(SD)	*P*-value^a^

Medically related absenteeism	31.5	(28.1)	25.3	(18.2)	0.0573
Disability	37.4	(75.4)	20.4	(50.8)	0.1048

Workloss costs	mean	(SD)	mean	(SD)	*P*-value^a^

Total employer costs	$11,298	($11,830)	$9,043	($12,416)	0.2161
Medically related absenteeism	$6,329	($7,136)	$5,687	($6,200)	0.5012
Disability	$4,970	($10,994)	$3,356	($10,284)	0.3134

SD indicates standard deviation.

^a^ *P*-values are determined using McNemar tests for proportions and paired *t*-tests for continuous measures.

^b^Workloss rate defined as the number of people with ≥1 disability claim or ≥1 instance of medically-related absenteeism.
